# The Expansion of Sirtuin Gene Family in Gilthead Sea Bream (*Sparus aurata*)—Phylogenetic, Syntenic, and Functional Insights across the Vertebrate/Fish Lineage

**DOI:** 10.3390/ijms25116273

**Published:** 2024-06-06

**Authors:** Paula Simó-Mirabet, Fernando Naya-Català, Josep Alvar Calduch-Giner, Jaume Pérez-Sánchez

**Affiliations:** Instituto de Acuicultura Torre de la Sal (IATS, CSIC), 12595 Ribera de Cabanes, Castellón, Spain; paula.simo@uca.es (P.S.-M.); fernando.naya@iats.csic.es (F.N.-C.); j.calduch@csic.es (J.A.C.-G.)

**Keywords:** gilthead sea bream, sirtuin, phylogeny, synteny, gene duplication, neo-functionalization, adaptive plasticity

## Abstract

The Sirtuin (SIRT1-7) family comprises seven evolutionary-conserved enzymes that couple cellular NAD availability with health, nutrition and welfare status in vertebrates. This study re-annotated the sirt3/5 branch in the gilthead sea bream, revealing three paralogues of *sirt3* (*sirt3.1a*/*sirt3.1b*/*sirt3.2*) and two of *sirt5* (*sirt5a*/*sirt5b*) in this Perciform fish. The phylogeny and synteny analyses unveiled that the Sirt3.1/Sirt3.2 dichotomy was retained in teleosts and aquatic-living Sarcopterygian after early vertebrate 2R whole genome duplication (WGD). Additionally, only certain percomorphaceae and gilthead sea bream showed a conserved tandem-duplicated synteny block involving the mammalian-clustered sirt3.1 gene (*psmd13*-*sirt3.1a/b*-*drd4*-*cdhr5*-*ctsd*). Conversely, the expansion of the Sirt5 branch was shaped by the teleost-specific 3R WGD. As extensively reviewed in the literature, human-orthologues (*sirt3.1*/*sirt5a*) showed a high, conserved expression in skeletal muscle that increased as development advanced. However, recent *sirt3.2* and *sirt5b* suffered an overall muscle transcriptional silencing across life, as well as an enhanced expression on immune-relevant tissues and gills. These findings fill gaps in the ontogeny and differentiation of Sirt genes in the environmentally adaptable gilthead sea bream, becoming a good starting point to advance towards a full understanding of its neo-functionalization. The mechanisms originating from these new paralogs also open new perspectives in the study of cellular energy sensing processes in vertebrates.

## 1. Introduction

Sirtuins (SIRTs) are a family of evolutionarily conserved enzymes that couple protein deacylation with the energy status of the cell via the cellular NAD^+^/NADH ratio [1]. These NAD^+^-dependent enzymes share different cellular locations (nucleus, cytoplasm, and mitochondria), and a wide tissue-specific gene expression pattern that reflects their involvement in a variety of fundamental biological processes [2,3,4]. Indeed, targets of SIRTs include regulators of DNA repair, inflammation, and energy metabolism [5]. In mammals, this protein family is composed of seven paralog counterparts (SIRT1-7), and their down-regulated expression has been associated with the pathophysiology of aging [6]. Certainly, there is now evidence that SIRTs regulate multiple processes associated with the pathogenesis of Alzheimer’s disease; therefore, the therapeutic activation of SIRTs is emerging as an active area of research in the field of age-related disorders in humans [7]. However, a comprehensive evolutionary survey of the SIRT family remains unclear, though a recent study recognized the expansion of “Class I SIRT (SIRT1-3)” and “Class IV SIRT (SIRT6 and 7)” around the origin of animal branching, raising up to nine the number of SIRT paralogs [8]. Moreover, recent studies have highlighted the occurrence of an additional SIRT isotype in the genome of non-amniote vertebrates (cartilaginous fish, bony fish, coelacanth, lungfish, and amphibians) that was annotated as Sirt3.2 [9]. However, a universal taxonomy assignment remains difficult, being of relevance to untangle the evolutionary history and functional shift of the SIRT/Sirt gene family across the evolution of the vertebrate lineage.

In gilthead sea bream (*Sparus aurata*), an extensively cultivated fish in the Mediterranean basin, transcriptional studies revealed a ubiquitous *sirt* gene expression that was tissue-specific for each *sirt* isotype [10,11,12]. According to this, *sirt1*, but also *sirt2* and *sirt5*, are expressed at relatively high levels. In contrast, *sirt3*, *sirt4*, *sirt6*, and *sirt7* have been categorized as *sirt* isotypes with relatively low transcriptional activity. The exon–intron organization is also specific to each *sirt* paralog, ranging the number of exons from 3 in *sirt4* to 16 in *sirt2*, though the gene organization (length and number of exons) seems to be relatively conserved for each *SIRT* ortholog through the vertebrate evolution. Conversely, the number and location of CpG islands (CGIs) are quite variable among fish genomes [13], and *sirt* genes in particular, despite their recognized role as an evolutionarily conserved mechanism that protects DNA from methylation, shaping the epigenome and the cell- and tissue-specific transcriptional patterns through development [14]. Thus, CGIs in close association with SP1 binding sites were predicted in the *sirt1* promoter of several fish, including gilthead sea bream (gene ID: 1847824780), fugu (gene ID: 101061405), zebrafish (gene ID: 797132), tilapia (gene ID: 100700447), and Atlantic salmon (gene ID: 106576833). However, the CGIs of the *sirt3* promoter do not appear to be conserved in all teleostean fish, which will be indicative of different permissive transcriptional rates, though it is not always true that genes with CGI promoters shaped a ubiquitous gene expression [15].

The aim of this study was to examine further the function and regulation of Sirt family in fish with a special focus on the farmed gilthead sea bream, a eurytherm, euryhaline, and protandrous hermaphrodite species with a well-recognized capacity to adapt to intensive aquaculture practices and alternative fish feeds [16,17,18,19,20]. The ultimate mechanisms responsive to this high functional plasticity remain elusive, though it can be favored by a high rate of short segmental duplications (SSDs) in a fish species with a third round (3R) of whole genome duplication (WGD) [21]. Certainly, WGD events result in hundreds to thousands of retained gene duplications, with great potential for the acquisition of new functions or sub-functionalizations of duplicates by the co-evolution of functionally related genes [22]. Indeed, the genome of gilthead sea bream (1.24 Gb) spans more than the genome of turbot (*Scophthalmus maxima*) (0.54 Gb) [23] or the European sea bass (*Dicentrarchus labrax*) (0.68 Gb) [24], but less than that of the common carp (*Cyprinus carpio*) (1.7 Gb) [25] and Atlantic salmon (*Salmo salar*) (2.90 Gb) [26] with a recent tetraploidization event (4R) [27]. The role of the components of the Sirt family could be of importance for the remarkable adaptive plasticity of this valuable cultured fish. Thus, we have re-examined the two already assembled gilthead sea bream genomes [21,28] in order to uncover new *sirt* isotypes, and to further classify and reannotate the components of this family by means of a combined approach based on structural genomics, phylogenetic, and synteny analyses. Furthermore, comprehensive transcriptomic profiling to identify the most abundant *sirt* isotypes per tissue was conducted. Finally, we monitored in fish development, from 60 days to 3 years of age, the expression patterns of relevant *sirt* isotypes and other syntenic genes focusing on insulin-like growth factor-binding proteins (*igfbp1*, *igfbp3*) in metabolically and growth-relevant tissues such as white skeletal muscle and liver. Altogether, this work represents an initial step in revealing how the adaptive expansion/contraction of some gene families such as *sirt* can contribute to increasing fitness and adaption through the evolution and in a challenging environment.

## 2. Results

### 2.1. Updated Sirt Catalogue

**Figure 1:**
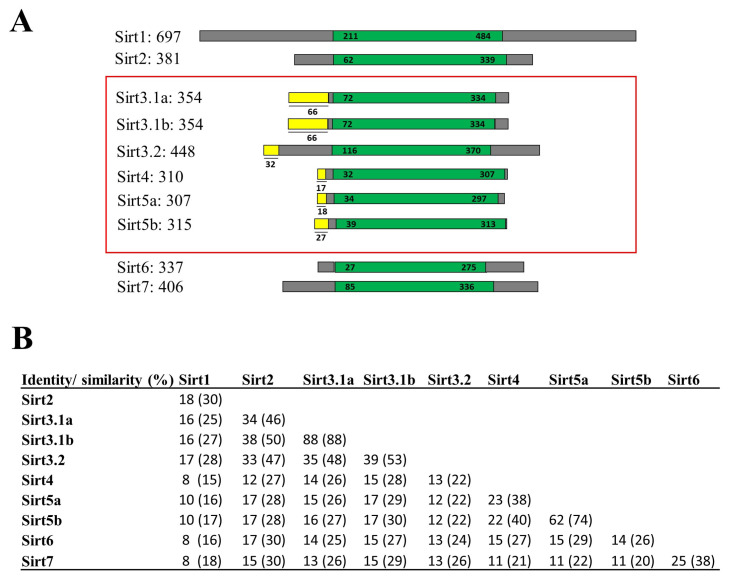
(**A**) Graphical representation of all gilthead sea bream Sirt proteins. The catalytic core domain (in green) and the surrounding N- and C-terminal regions (in grey) are represented. The mitochondrial target peptides of mitochondrial Sirts (framed in red) are indicated in yellow. Numbers correspond to amino acid residues. Accession numbers of genes are listed below: *sirt3.1a* (OR394775; XP_030270596.1), *sirt3.1b* (OR394776), and *sirt3.2* (AHX56275); human *SIRT5* (Gene ID: 23408); GSB *sirt5a* (AHX56277); and *sirt5b* (OR394777). (**B**) Percentages of amino acid sequence identity and similarity (in parentheses) among gilthead sea bream Sirt family members.

Data from PhylomeDB evidenced up to three new gilthead sea bream *sirt* gene isotypes that were embraced by SIRT3 and SIRT5 branches (Figures S1 and S2) (http://phylomedb.org (accessed on 23 May 2022); PHY00CLOW5_SPAAU and PHY00CKZTK_SPAAU accession numbers). These new sequences were uploaded to GenBank with accession numbers OR394775 (*sirt3.1a*), OR394776 (*sirt3.1b*), and OR394777 (*sirt5b*), being renamed their gilthead sea bream paralogs as *sirt3.2* (AHX56275, before *sirt3*) and *sirt5a* (AHX56277, before *sirt5*) based on sequence, phylogeny, and synteny analyses. The graphical representation of all gilthead sea bream Sirt proteins with data on amino acid sequence identity and similarity are shown in Figure 1. A conserved catalytic domain of about 250 amino acids in length was evidenced in all the reported sequences, and mitochondrial peptide targets of 17–66 amino acids in length were identified in Sirt3, Sirt4, and Sirt5 paralogs (Figure 1A). According to the current SIRT classification of [29], the highest sequence conservation was found between Sirt1-3 (Class I), as well as Sirt4 (Class II) and Sirt5 (Class III), and Sirt6 and Sirt7 (Class IV). This rendered amino acid sequence similarities of 53–99% and 74% for the comparisons made within the expanded gene families of Sirt3 and Sirt5, respectively (Figure 1B).

### 2.2. Gene Structure and Organization

**Figure 2:**
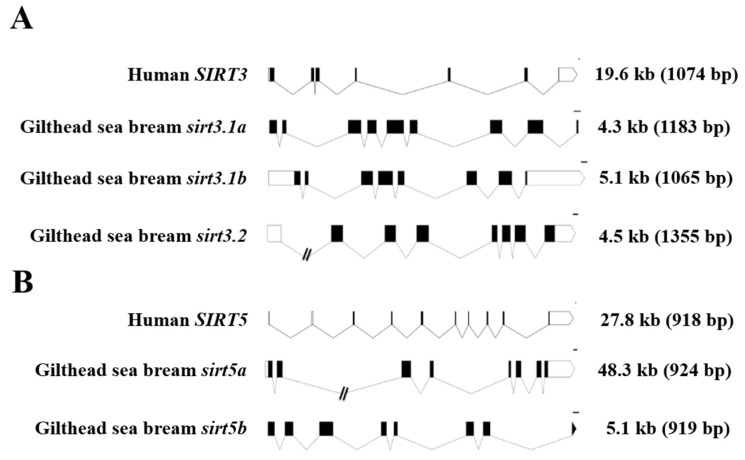
Schematic representation of the exon–intron structure of the SIRT3 (**A**) and SIRT5 (**B**) paralogs of gilthead sea bream and human. White and black boxes represent the noncoding and coding exons, respectively. Introns are shown as connecting lines. Scale bars are 100 bp. Numbers indicate the total length of the sequences from ATG to the stop codon including and excluding (in brackets) introns.

For comparative purposes, the exon–intron organization of human and gilthead sea bream *SIRT3/sirt3* and *SIRT5/sirt5* genes is shown in Figure 2. As a general feature, the retrieved gilthead sea bream *sirt3* genes (*sirt3.1a*, *sirt3.1b*, *sirt3.2*) were shorter than the human *SIRT3* counterpart (4.3–5.1 kb vs. 19.6 kb), changing the number of protein-coding exons from seven in the human *SIRT3* to eight to nine in the duplicated gilthead sea bream *sirt3.1* gene (Figure 2A). In contrast, the human and gilthead sea bream *SIRT5/sirt5* genes showed the same number of protein-coding exons (8), but the gene length varied from 48.3 kb in *sirt5a* to 27.8 kb in *SIRT5*, and 5.1 kb in *sirt5b* (Figure 2B), conserving, more accurately than *SIRT3/sirt3*, the length of their coding region.

### 2.3. Phylogenetic and Syntenic Analyses

**Figure 3:**
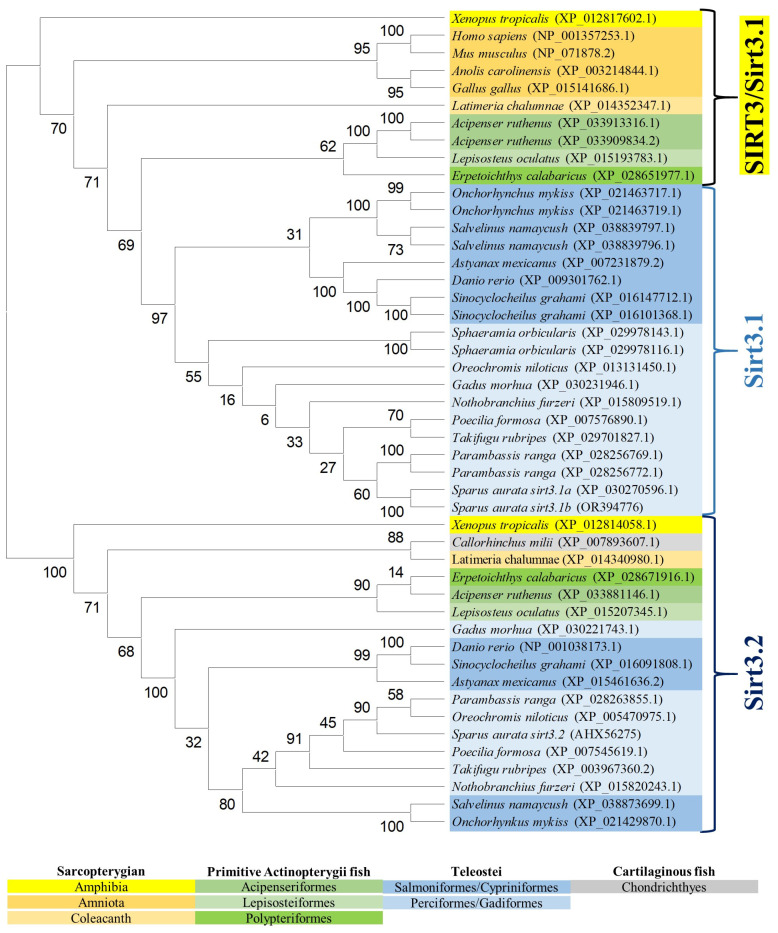
Phylogenetic tree of vertebrate SIRT3, generated by maximum likelihood algorithm in Mega software version 11. The sequences included in the analysis correspond to full protein sequences from 23 vertebrate species. The accession numbers from GenBank, OrthoDB, and IATS-CSIC gilthead seabream genome are included in brackets. Numbers in branches indicate the bootstrap confidence in the resulting phylogenetic tree measured by auto-bootstrapping mode.

**Figure 4:**
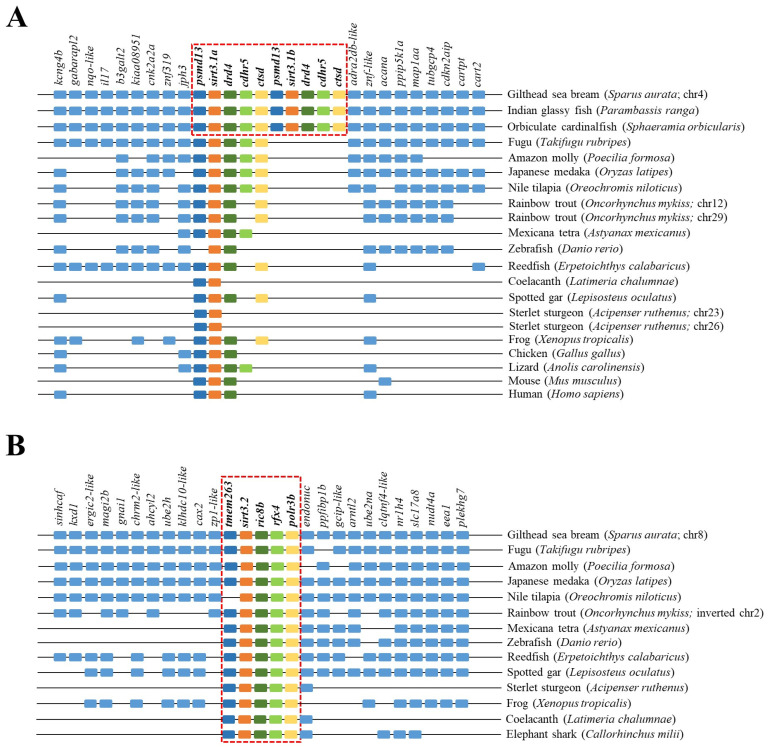
Gene synteny of *sirt3.1a/b* (**A**) and *sirt3.2* (**B**) in different vertebrate species. The synteny was analyzed with Genomicus v100.01 using the gene orders of gilthead seabream as reference. Orthologs of *sirts* in other species are shown in matching colors. A line between two genes is equivalent to a break in the continuity of the alignment. A red discontinuous square corresponds to the conserved syntenic block in different species.

The reconstructed phylogeny of the SIRT3 family evidenced two main branches according each one with the present hierarchy of vertebrates (Figure 3). The branch named SIRT3/Sirt3.1 embraced the sarcopterygians (amniotes, amphibian, coelacanth), and the actinopterygians (ray-finned fish) that disclosed separately the node of primitive actinopterygii fish (polypteriformes/acipenseriformes/lepisosteiformes) and that of modern fish of the infra-class teleostei represented in the branch Sirt3.1. This last taxonomic group also disclosed two main monophyletic groups corresponding to Salmoniformes/Cypriniformes and Perciformes/Gadiformes, showing most species of this fish lineage duplicated *sirt3.1* genes as a result of whole or segmental tandem genome duplications. In contrast, the node named Sirt3.2 was a long branch that encompassed a single copy of *sirt3* paralogs from cartilaginous fish (chondrichthyes), lobe-finned fish (coelacanthiformes), and aquatic tetrapods (frogs) to primitive and modern fish (actinopterygii). The synteny analyses also rendered a different genome cartography for each one of these two SIRT3/Sirt3 branches. Thus, *sirt3.1* has evolved in gilthead sea bream as a duplicated synteny block of five genes (*psmd13*/*sirt3.1*/*drd4*/*cdhr5/ctsd*) located in the super-scaffold/chromosome 4 (Figure 4A). This segmental tandem genome duplication also occurred in other percomorphaceae (*Parambassis ranga*, *Sphaeramia orbicularis*), and it persisted through the evolution of sarcopterygians as a well conserved *psmd13*/*sirt3.1/drd4* single-synteny block. Likewise, the branch of Sirt3.2 evolved across amphibians, cartilaginous fish, and the actinopterygian fish class as a highly conserved single-synteny block (*tmem263/sirt3.2/ric8b/rfx4/polr3b*) that is located in the super-scaffold/chromosome 8 of gilthead sea bream (Figure 4B).

**Figure 5:**
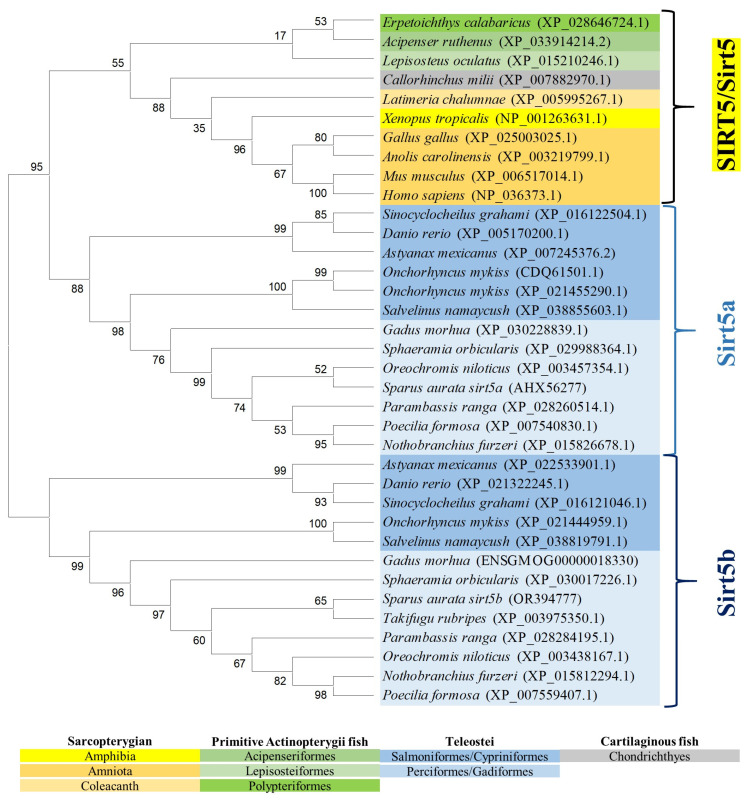
Phylogenetic tree of vertebrate SIRT5, generated by maximum likelihood algorithm in Mega 11 software. The sequences included in the analysis correspond to full protein sequences from 23 vertebrate species. The accession numbers from GenBank, OrthoDB, and IATS-CSIC gilthead seabream genome are included in brackets. Numbers in branches indicate the bootstrap confidence in the resulting phylogenetic tree measured by auto-bootstrapping mode.

**Figure 6:**
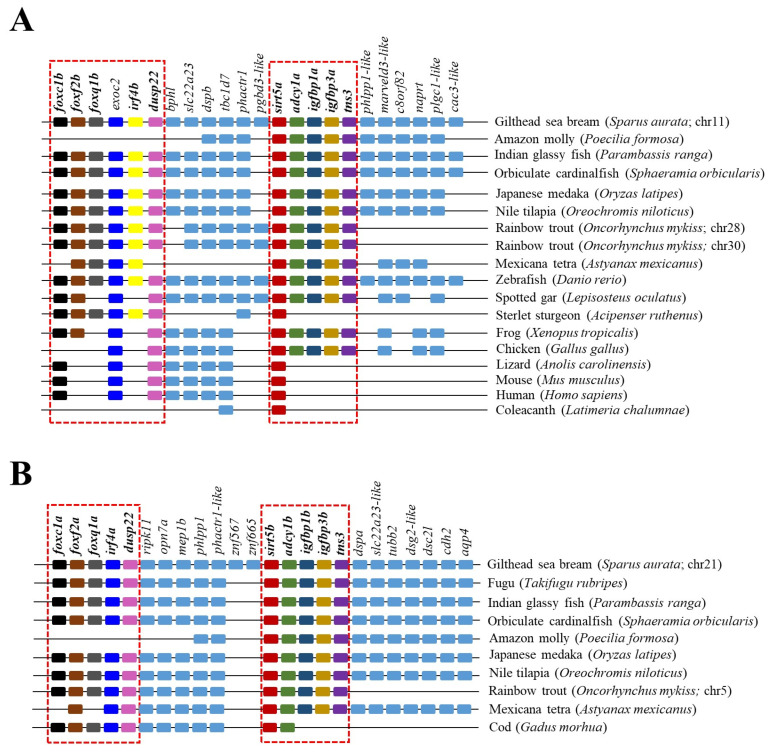
Gene synteny of *Sirt5a* (**A**) and *Sirt5b* (**B**) copies in different vertebrate species. The synteny was analyzed with Genomicus v100.01 using the gene orders of gilthead seabream as reference. Orthologs of *sirts* in other species are shown in matching colors. A line between two genes is equivalent to a break in the continuity of the alignment. The red discontinuous square corresponds to the conserved syntenic blocks in different species.

The reconstructed phylogenetic tree of SIRT5 also rendered a long branch that separately embraced sarcopterygians, primitive fish, and teleosts, according to the present hierarchy of vertebrates with the exception of chondrichthyes that were more related to sarcopterygians rather than to bony fish (Figure 5). On the other hand, most primitive fish (acipenseriformes/lepisosteiformes/polypteriformes) evolved as a main outgroup that constitutes, together with sarcopterygians, the clade SIRT5, whereas the teleostei disclosed two additional nodes (Sirt5a, Sirt5b) due to the conservation of two gene copies of *sirt5* (super-scaffold/chromosome 11 and 21 in gilthead sea bream) in almost all the members of this fish lineage. The analysis of synteny also supported this dichotomous trend with two highly conserved synteny blocks (*foxc1/foxf2/foxq1/irf4/dusp22*; *adcy1/igfbp1/igfbp3/tns3*) in each one of these two teleostean *Sirt5a*/*Sirt5b* nodes (Figure 6). One or both of these gene synteny blocks were lost in the primitive actinopterygii (Sterlet sturgeon) and sarcopterygian fish (coelacanth), being weakened through the evolution of tetrapods (except in *Gallus gallus* and *Xenopus tropicalis*), which becomes especially evident for the conserved order of aligned genomic blocks of *sirt5b* (Figure 6B).

### 2.4. Tissue-Specific Gene Expression

**Figure 7:**
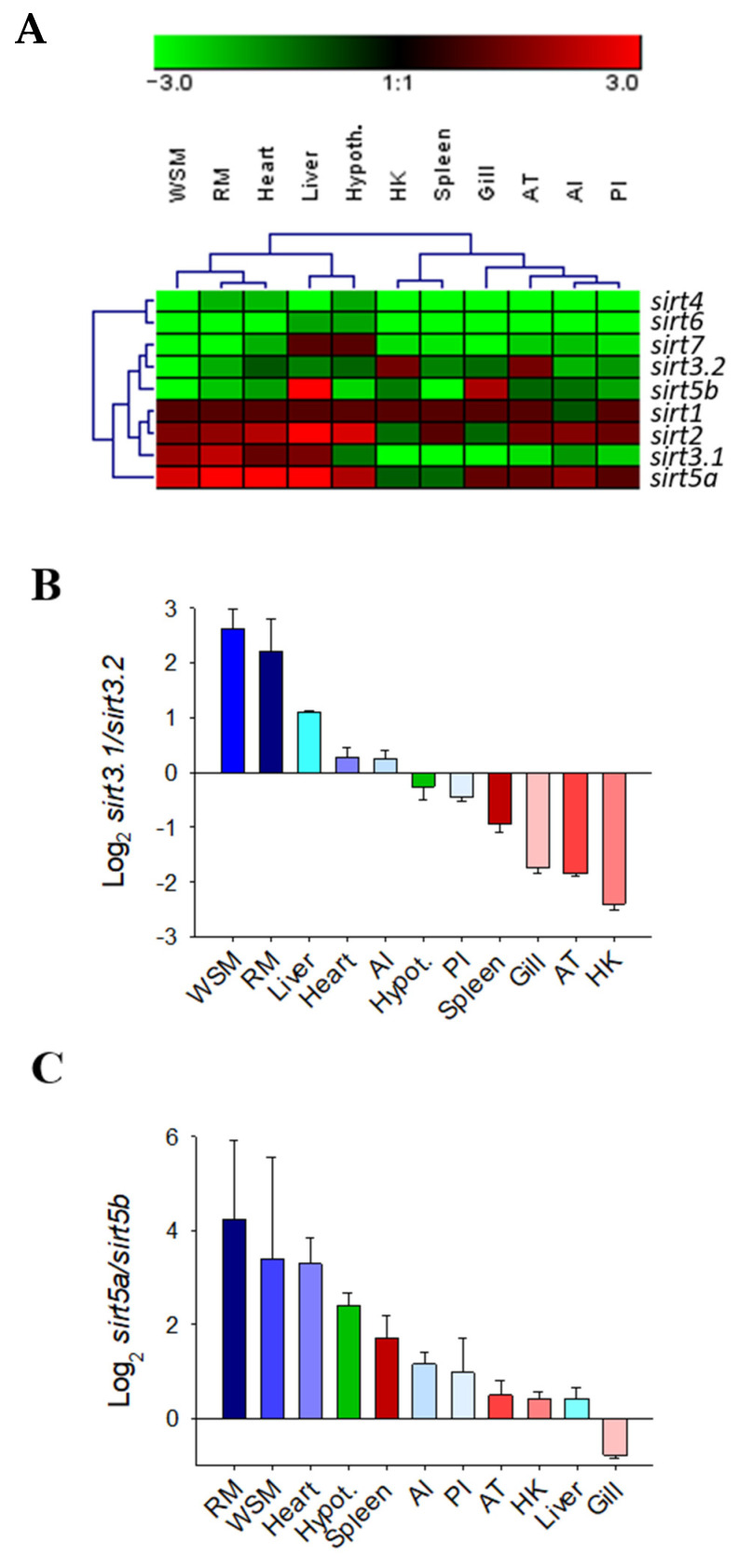
Hierarchical heatmap of the gene expression pattern of *sirts* in 11 tissues with different metabolic capabilities (**A**). Ratio between *sirt3.1* and *sirt3.2* (**B**) or *sirt5a* and *sirt5b* (**C**) gene duplications in 11 tissues of gilthead sea bream.

The gene expression pattern of the complete catalogue of gilthead sea bream *sirts* was assessed in a wide range of tissue of adult fish with different metabolic and physiological features (Figure 7A). Specific PCR primers were designed for each *sirt* paralog, with the exception of *sirt3.1* primers that amplified both the *sirt3.1a* and *sirt3.1b* transcripts due to the difficulty in designing specific primers for each one of these duplicated genes (codifying sequence, 99% nucleotide identity, see Figure 1B). Overall *sirt1*, *sirt2*, and the now named *sirt5a* and *sirt3.1* were the most ubiquitously expressed *sirts* across all the analyzed tissues; meanwhile, low expression levels were reported for *sirt4* and *sirt6* in almost all the analyzed tissues. Relatively low expression levels were also disclosed for *sirt7* in almost all tissues, except for the liver and hypothalamus. The same for the novel *sirt5b*, which only shared relatively high levels of expression in liver and gills in comparison to the other *sirt* counterparts. Likewise, the now named *sirt3.2* showed a relatively high expression level in head kidney (HK) and adipose tissue (AT), whereas the *sirt3.1* paralogs (*sirt3.1a* + *sirt3.1b*) displayed high expression levels in liver and muscle tissues (white skeletal muscle, WSM; red muscle, RM; and heart). On closer examination, the different expression pattern of duplicated genes of *sirt3* and *sirt5* paralogs is disclosed as a quotient expression ratio of *sirt3.1/sirt3.2* and *sirt5a/sirt5b* (Figure 7B,C).

### 2.5. Differential Expression Patterns of Sirts and Syntenic Igfbps across Development

Gene expression across development with whole larvae, WSM, and the liver as targeted tissues was focused on *sirt1*, *sirt2*, *sirt3*, *sirt5*, and synteny *igfbp* genes of *sirt5a* (*igbp1a*, *igbpb3a*) and *sirt5b* (*igfbp1b*, *igfbp3b*). As shown in Table 1, the expression level of all the analyzed genes was changed through the development. As a general feature, the expression level of *sirt3.1* increased consistently with advancing age. In contrast, the expression levels of *sirt1*, *sirt3.2*, and *sirt5b* decreased markedly. Less clear is the pattern in fish development of *sirt2* and *sirt5a*, which achieved the maximum expression level in three-year-old fish after an invariable or decreasing trend during early life stages. This also resulted in a pronounced increase in the expression ratio of *sirt3* (*sirt3.1/sirt3.2*) and *sirt5* (*sirt5a/sirt5b*) paralogs in advanced development. In parallel, the expression level of *igfbps* was markedly decreased *(igfbp1a*) or almost silenced *(igfbp1b*, *igfbp3b*) through the development, whereas a pronounced up-regulation was evidenced in the case of *igfbp3a.* In comparison to WSM, the developmental changes in the hepatic gene expression signature were less pronounced (Table 2). In any case, the hepatic expression of *sirt1* and *sirt3.2* was decreased significantly through development, which in turn increased the expression ratio of *sirt3.1/sirt3.2*. In parallel, the hepatic gene expression of *igfbp1* paralogs (*igfbp1a*, *igfbp1b*) decreased with advancing age, but always remained detectable with a relatively high level of expression in the case of *igfbp1b*. In contrast, the gene expression of *igbp3* paralogs (*igfbp3a*, *igfbp3b*) was almost silenced through the development in the liver tissue.

## 3. Discussion

SIRTs build a gene family of deacylases with a complex evolutionary history across eukaryotic organisms that makes it difficult to establish a common SIRT repertoire; however, at least seven paralogs can be traced back to the last common ancestor of vertebrates [9]. The present study confirmed this statement, increasing the number of Sirt family members from seven to ten in a Perciform fish, the gilthead sea bream (Figure 1A), which served to disclose up to three gene copies of *sirt3* (*sirt3.1a*, *sirt3.1b*, *sirt3.2*) and two copies of *sirt5* (*sirt5a*, *sirt5b*). All of them were nuclear-encoded mitochondrial proteins as evidenced in the conservation of a mitochondrial targeting sequence of 17–66 amino acids in length that would direct the newly synthetized proteins towards the mitochondria (Figure 1A). From an evolutionary perspective, phylogenomic approaches indicate that the mitochondrial proteome expanded through vertebrate evolution not only by gene/genome duplications of mitochondrial proteins, but also by the re-localization of the paralogs to the organelle in a tissue-specific manner [30]. The most common process is the intra-mitochondrial duplication, but regardless of this, the establishment of new gene balances is always challenging, and the polyploids that survive a WGD event typically undergo a rediploidization process that leads to genome fractionation. Conversely, the preservation of duplicated genes can signify an increase in its activity (gene dosage), a novel acquired function (neo-functionalization), or the division among the copies of the functions of the ancestral gene (sub-functionalization) [31]. This is the basis of the evolution of the teleostean lineage that experienced a third WGD event (3R), followed by a 4R WGD in the branch belonging to the modern lineage of cyprinids and salmonids [27,32,33]. Conversely, alternative splicing arises in terrestrial vertebrates as the preferred mechanism for increasing gene diversity [34,35]. Thus, recent human genomic searches disclosed up to 38 and 43 protein isoforms of SIRT3 (https://www.ncbi.nlm.nih.gov/gene/23410; last accessed: 17 January 2024) and SIRT5 (https://www.ncbi.nlm.nih.gov/gene/23408; last accessed: 17 January 2024), respectively. These alternative variants often show a tissue-specific gene expression pattern, extending the way in which mitochondrial, cellular, and organism homeostasis can be regulated in an effective manner [36,37]. Certainly, meta-analytical approaches have identified the mitochondria as a key player in stress adaptive responses in a wide range of living organisms, including fish [38]. With these considerations in mind, we aimed to better understand the evolution and functional significance of the expanding Sirt3 and Sirt5 family in a farmed fish model, with a well-recognized predisposition of gene expansion [21,39]. 

On closer examination, phylogenetic attempts disclosed two SIRT3 orthologs (2R WGD) at the early vertebrate evolution [8] with vestiges remaining in the genome of the modern sarcopterygians (Figure 3) that support the Sirt3 division into Sirt3.1/Sirt3.2 in non-amniote aquatic-living vertebrates [9]. The Sirt3.1 branch also discerned *sirt3.1a* and *sirt3.1b* duplicates in gilthead sea bream and in two other percomorphaceae (*S. orbicularis*, *P. ranga*), which might indicate a gene retention after the 3R WGD (Figure 3). However, these paralogs accumulated very low intra-sequence polymorphisms (Figure 1B), which can lead to theories that the *sirt3.1* duplication had occurred recently in the evolution [40]. This is further supported by the conservation in these fish species of a duplicated tandem repeat of five genes (*psmd13*-*sirt3.1a/3.1b*-*drd4*-*cdhr5*-*ctsd*) in each one of these two *sirt3.1* paralogs (Figure 4A), which differ largely from the synteny block (*tmem263/sirt3.2/ric8b/rfx4/polr3b*) of the *sirt3.2* paralog retained through the evolution as a unique gene copy (Figure 4B).

The importance of SIRT3 in metabolic homeostasis is widely documented in the skeletal muscle of humans and rodents, in which the TRF2–SIRT3 axis connects telomere shortening with muscle adaptive metabolism, development, and aging [41,42]. Thus, not surprisingly, TRF2 (telomeric repeat-binding factor 2) ablation in mouse skeletal muscle leads to *Sirt3* down-regulation, highlighting the functional importance of the TRF2-mediated chromatin loop in regulating *Sirt3* gene expression, and subsequently oxidative metabolism and cellular senescence. Mechanistically, most of these processes remain elusive, though it is known that the anti-aging effects of the adjudin drug are exerted by elevating the expression level of *SIRT3*, which in turn reduces cellular levels of reactive oxygen species (ROS) by deacetylating forkhead box O3a (FOXO3a), a transcription factor that transactivates antioxidant genes, such as catalase (CAT) and manganese superoxide dismutase (SOD2) [43,44]. In agreement with this, in gilthead sea bream, the expression level of *sirt3.1* reached its highest expression level in RM and WSM (Figure 7B), with an enhanced expression through development in the case of WSM (Table 1 and Table 2) that becomes largely constitutive in the liver (Table 2). Otherwise, it must be noted that the *sirt3.1* gene lies very close to *pmsd13* (proteasome 26S subunit, non-ATPase 13) through almost all the vertebrate evolution (Figure 4A), with the occurrence in humans of a bidirectional promoter that reinforces the link of metabolic condition and mitochondrial and cellular stress responses with the proteolysis of unfolded proteins [45,46]. If this is the case in other organisms, such an observation gives support to a subrogate marker of deviations in biological age in a wide range of living organisms, including farmed fish. In order to ensure this hypothesis, further research becomes necessary for determining the effect of ecological and physiological factors over this gene.

Unlike *sirt3.1*, both in this (Figure 7B) and a previous study [10], the regulation of *sirt3.2* was driven towards an active gene expression in mucosal and immuno-relevant tissues of gilthead sea bream (HK, spleen, gills, intestine), which was concurrent herein with a developmentally regulated gene silencing in both liver and WSM (Table 1 and Table 2), also reported for the ortholog of *Xenopus tropicalis* (https://www.bgee.org/gene/ENSXETG00000014800; last accessed: 17 January 2024; [47]) (Figure 4B). Since SIRTs are considered to have anti-inflammatory properties due to their regulatory effects on several transcription factors and their downstream pro-inflammatory effectors [48], such a gene expression pattern can be viewed as a negative feedback regulation of inflammation. Certainly, SIRTs are highly activated during a number of conditions known to enhance NAD^+^ bioavailability including nutrient restriction (fasting), exercise, and late acute inflammation. On the contrary, aging, nutrient overload, and activation of the hypoxia–ROS–early inflammation triad lead to decreased cellular NAD^+^ levels, which translate into the reduced gene expression and enzymatic activity of SIRTs. This was supported by recent observations in gilthead sea bream, where the ectoparasite *Sparicotyle chrysophrii*, parasitizing the gill epithelium, triggered the up-regulation of apoptotic markers in combination with a down-regulation of *sirt3.2* and hypoxia-related genes [49]. Likewise, also in gilthead sea bream, there is now experimental evidence that high stocking density and mild-hypoxia pre-conditioning down-regulated *sirt3.2* expression in the heart [50] and WSM [18]. Altogether, the interest in targeting *sirt3* on livestock-fish-farming and its function acquisition in the context of climate change with an increase in water temperature and reduced oxygen availability merits further research.

As reported for SIRT3/Sirt3, the SIRT5/Sirt5 branch also expanded through the evolution of vertebrates, rendering two Sirt5 isotypes that remained conserved in the lineage of the modern teleost as two separated Sirt5a and Sirt5b clusters (Figure 5). The Sirt5a branch was phylogenetically closer to its human ortholog, forming a conjoint clade composed of teleost and non-teleost organisms. In contrast, the Sirt5b branch was exclusive to bony fish and persisted as a separate node with no vestiges of it in sarcopterygians. This, together with a relatively low phylogenetic divergence of Sirt5a/Sirt5b (74% amino acid similarity) in comparison to Sirt3.1/Sirt3.2 (53% amino acid similarity), supported the acquisition of a novel Sirt5b isotype as a teleost-specific Sirt after the 3R WGD event of teleosts, rather than a duplication event at the early vertebrate evolution (2R WGD). In addition, the *sirt5a* was expressed more actively than *sirt5b* across a large range of tissues, with the exception of the gills where the expression of *sirt5b* was at least two-fold higher than that of *sirt5a* (Figure 7). If this is indicative of a tissue-specific activity related to aquatic life remains elusive, though it has been suggested that the Sirt5 counterpart of marine mussels would drive different cellular stress responses to alleviate signs of heat stress [51]. In particular, in gilthead sea bream, *sirt5a* becomes expressed actively in both anaerobic (WSM) and aerobic (RM, heart) muscle tissues. Furthermore, the expression level of *sirt5a* was maintained high in WSM through development, whereas it was almost suppressed in the case of *sirt5b* a few days after hatching. The close association of *sirt5a* and muscle growth and metabolism was also reported in previous gilthead sea bream studies, in which growth suppression with fasting [10] and advancing age [15] was associated with a pronounced increase in *sirt5a* gene expression. In both cases, this occurred in coincidence with a reduced energy wastage, evidenced by the down-regulation of muscle mitochondrial uncoupling proteins and changes in the gene expression patterns of markers of lipid metabolism and oxidative phosphorylation, according to the general idea that SIRTs restrain any energy-consuming cellular activity, including growth and inflammation, until the abatement of metabolic stress disturbances [48,52]. 

Early studies have also supported a crosstalk between SIRTs and the GH/IGF axis, a key endocrine system regulating growth in vertebrates. Attention has been focused on SIRT1, and studies in mice have evidenced that the in vivo knockdown of hepatic *SIRT1* restores the fasting-induced decrease in circulating levels of IGF-I [53]. Additionally, SIRT1 acts at the brain level as a link between somatotropic signaling and calorie restriction [54], and brain SIRT1 knockouts displayed dwarfism and reduced plasma GH and IGF-I levels [55]. According to this, SIRT1 activation contributes to suppress the GH/IGF tonus, which would serve to drive a decreased supply of energy for growth purposes in a cellular milieu with a reduced availability of metabolic fuels [53]. The relationship of the GH/IGF axis with other SIRT paralogs remains mostly understood, but intriguingly, our synteny analysis highlighted a high conservation through all the vertebrate evolution of a genomic synteny block with SIRT5 and IGBP1/3 as neighboring genes (Figure 6). This offers the possibility of the maintenance of genomic regulatory blocks, where the regulatory domain of a regulatory gene can extend into and beyond adjacent transcriptional units to shape the co-regulated expression of neighboring genes [56,57]. Focusing on IGFBPs, it is now recognized that the ancestral IGFBP gene was duplicated at an early animal stage to produce a pair of IGFBPs that gave rise in subsequent duplication events to the two IGFBP clades in modern vertebrates (IGFBP1/2/4; IGBP3/5/6), with a differentially regulated expression in liver and skeletal muscle that is accomplished early in the development of gilthead sea bream [18,52]. In adult fish, such a regulatory feature rendered a clear dominance of the *igbp3a* expression in the WSM of adult fish, which might depict a co-regulated expression of *sirt5a* and *igbp3a* as a synteny block in this muscle tissue (Table 1). Conversely, the silencing of *sirt5b* should be shaped by a genomic regulatory block involving the co-regulated inhibition of *igfbp1b*. However, less clear are these types of associations at the liver level, in which other regulatory mechanisms might serve to preserve a more constitutive gene expression of all *sirt3* and *sirt5* paralog pairs (Table 1 and Table 2).

In summary, this research highlighted the expansion of the Sirt3 and Sirt5 family in gilthead sea bream at different times through evolution, increasing the repertoire of Sirt genes from seven to nine in our farmed fish model. As their mammalian SIRT counterparts, the named *sirt3.1a/b* and *sirt5a* were mostly expressed at a relatively high level in muscle tissues, with perhaps a different contribution to proteolysis of unfolded proteins and muscle growth regulation via the Gh/Igf system, as inferred from the gene synteny block analysis. In contrast, the named *Sirt3.2* evolved as an exclusive isotype of aquatic organisms with a mainly envisaged immunoregulatory role based on gilthead sea bream tissue-specific gene expression patterns. Likewise, *Sirt5b* emerged as an exclusive teleostean Sirt isotype with an enhanced expression in gills that might support some specific adaptive features like osmoregulation and heat stress. Overall, these findings confirm this first approach into the origin and evolution of the sirtuin family in gilthead sea bream. However, although gene expression data included in this analysis provide crucial insights into the potential tissue- and developmental-specialization of Sirts, it represents just one layer of the complex regulatory networks in living organisms. Integrating gene expression data with additional molecular, cellular, and phenotypic information is further required for a comprehensive understanding of the adaptive strategies of fish across development and in response to environmental challenges, reinforcing the importance of gene duplication shaping the landscape of the adaptable gilthead sea bream.

## 4. Materials and Methods

### 4.1. Ethics Statement

All procedures involving experimental animals were approved by the Ethics and Animal Welfare Committee of IATS, CSIC Ethics Committee (permission 1295/2022) and Generalitat Valenciana (permission 2022-VSC-PEA-0230). Such interventions were carried out in a registered installation facility (code ES120330001055) in accordance with the principles published in the European Animal Directive (2010/63/EU) and Spanish laws (Royal Decree RD53/2013) for the protection of animals used in scientific experimentation.

### 4.2. Fish Husbandry and Tissue Sampling

Fish were raised from early life stages to harvest with commercial pellets (0.2–0.8 mm Skretting, Burgos, Spain; 1.5–6.5 mm Biomar, Burgos, Spain) at the indoor experimental facilities of the Institute of Aquaculture Torre de la Sal (IATS, CSIC) under natural photoperiods and temperature conditions at our latitude (40°5 N; 0°10 E). Feed was offered daily near to visual satiety or on alternate days (3–7 times per week) depending on season and fish size. Water temperature ranged from 10 °C in winter to 27 °C in summer. The water oxygen concentration was always higher than 75% saturation, and unionized ammonia remained below toxic levels (<0.02 mg/L) irrespective of season. For the tissue-specific gene expression profiling, one-year-old fish (120 g average body weight) were fasted overnight in summer and representative tissues (liver, white skeletal muscle, red muscle, heart, gills, perivisceral adipose tissue, anterior intestine, posterior intestine, head kidney, spleen, and hypothalamus) with different metabolic capabilities were rapidly excised from five individuals, frozen in liquid nitrogen, and stored at −80 °C until RNA extraction. Additionally, six samples of whole body (larval stages), liver, and white skeletal muscle were taken through fish development (May–July) at 60-, 81-, and 127-days post-hatching (dph) from individuals with an averaged body weight of 137 mg, 1 g, and 4 g, respectively. At the last sampling point, liver and muscle tissues were also taken from 9–10 individual fish of 100 g (one-year old fish) and 1 kg (three-year old fish).

### 4.3. Phylogenetic and Synteny Analyses

The reconstructed gilthead sea bream PhylomeDB with protein-coding sequences from the IATS-CSIC assembled genome [21] was interrogated for *sirt* paralogs. This rendered up to three new sequences that were recognized as *sirt3* and *sirt5* gene isotypes that were manually curated by homology searches in the genome draft of [28]. This was followed by extensive searches for orthologs of *Sirt3/Sirt5* genes across the vertebrate lineage in the OrthoDB v11 database [58]. Multiple sequence alignments and similarity/identity calculations were carried out using GeneDoc v2.7 [59]. Mitochondrial target sites and *sirt* domains were predicted using the TargetP-2.0 (https://services.healthtech.dtu.dk/services/TargetP-2.0/, accessed on 23 November 2023) and PROSITE-Expasy (https://prosite.expasy.org/, accessed on 23 July 2023) online tools, respectively. Separate phylogenetic trees for SIRT3/Sirt3 and SIRT5/Sirt5 clades were constructed with the maximum likelihood algorithm in MEGA v11 [60], using JTT [61] substitution matrix based on the lowest Bayesian information criterion (BIC) score. Non-uniformity of evolutionary rates among sites were modeled by using a discrete Gamma distribution (+G) and bootstrap values were calculated by auto-bootstrapping method. All positions containing gaps and missing data were eliminated (complete deletion). For syntenic studies, neighboring genomic regions of *SIRT3*/*sirt3* and *SIRT5*/*sirt5* paralogs were established by querying the IATS-CSIC genomic database and the Genomicus tool v100.01 [62]. The syntenic blocks were the result of gene order arrangements from representative species of sarcopterygians (order coelacanthiformes and Superclass tetrapoda) and actinopterygians (Chondrostei and Neopterygii Subclasses). The lineage-representative group of species used in each approach, as well as their corresponding NCBI protein ID, can be found in Table S1. The graphical representation of gene organization was carried out with the online tool Exon-Intron Graphic Maker (https://wormweb.org/exonintron, accessed on 5 June 2023). The manuscript followed the ZFIN Zebrafish Nomenclature Guidelines for gene and protein names and symbols, with a/b assigned suffixes based on the suffix of the already annotated surrounding genes (https://wiki.zfin.org/display/general/ZFIN+Zebrafish+Nomenclature+Conventions, accessed on 3 February 2023).

### 4.4. Gene Expression Profiling

#### 4.4.1. RNA Extraction 

RNA from individual samples was extracted using the MagMAX-96 total RNA isolation kit (Life Technologies, Carlsbad, CA, USA). The RNA yield was 50–100 μg, with absorbance ratios (A260/A280) of 1.9–2.1. The RNA integrity number (RIN) values of 8–10 (Agilent 2100 Bioanalyzer; Agilent, Santa Clara, CA, USA) were indicative of clean and intact RNA.

#### 4.4.2. cDNA Synthesis

Reverse transcription of 500 ng of total RNA was performed with random decamers using a High-Capacity cDNA Archive Kit (Applied Biosystems, Foster City, CA, USA). Negative control reactions were run without reverse transcriptase. 

#### 4.4.3. qPCR-Array Setup

Different 96-well PCR arrays were designed for the simultaneous gene expression profiling of gilthead sea bream *sirts* and syntenic *igfbp* genes (*igfbp1a*, *igfbp1b*, *igfbp3a*, *igfbp3b*). Two housekeeping genes (β-actin and 18S rRNA) and controls of PCR performance were included in each array. In brief, 660 pg of total cDNA was used in 25 μL PCR reactions. PCR wells contained 2x SYBR Green Master Mix (Bio-Rad, Hercules, CA, USA) and specific primers at a final concentration of 0.9 μM (Table S2). All pipetting operations for the PCR arrays were performed by an EpMotion 5070 Liquid Handling Robot (Eppendorf, Hamburg, Germany) to improve data reproducibility. Real-time quantitative PCR was carried out in an Eppendorf Mastercycler Ep Realplex (Eppendorf, Hamburg, Germany). The PCR amplification program consisted of an initial denaturation step at 95 °C for 3 min, followed by 40 cycles of denaturation for 15 s at 95 °C, and annealing/extension for 60 s at 60 °C. The efficiency of the PCR reactions was consistently higher than 90% and was similar among all the genes. The specificity of the reactions was verified by melting curve analysis (ramping rates of 0.5 °C/10 s over a temperature range of 55–95 °C). Negative controls without a template were performed for each primer set. Gene expression was calculated using the delta-delta Ct method [63]. For multigene analysis, all values for whole larvae and muscle tissue were referenced to the expression level of *sirt1* in whole larvae (60 dph). Liver gene expression levels were referenced to the expression of *sirt1* at 81 dph, for which a value of 1 was arbitrarily assigned.

### 4.5. Updated Sirt Catalogue

Hierarchical clustering was performed using the Genesis software v9.1 [64] to assess the tissue-specific gene expression pattern of the complete catalogue of *sirt* genes in gilthead sea bream. Statistical analyses were performed using SigmaPlot version 14.0 (Systat Software, San Jose, CA, USA) with all *p*-values set to 0.05. Normality and equal variance of data were tested using Shapiro–Wilk and Levene tests, respectively. Developmental and tissue-specific differences in gene expression were analyzed by one-way ANOVA followed by Holm–Sidak test.

## Figures and Tables

**Table 1:** Relative gene expression of whole body (60 days post-hatching, dph) and white skeletal muscle (WSM) of gilthead sea bream across the development process. Data are the mean ± SEM of 6–10 fish. Data values are referenced to the expression level of *sirt1* in whole larvae (60 dph), for which a value of 1 was assigned. *p*-values are the result of one-way analysis of variance and different superscript letters indicate significant differences (Holm–Sidak test, *p* < 0.05, bold). For each gene, gradation of black filling in circles indicates the expression levels across the development process.

		Whole Body		WSM							
		60 dph		81 dph		127 dph		1 Year		3 Years	*p*-Value
***sirt1***	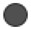	1.01 ± 0.07 ^a^	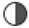	0.70 ± 0.05 ^b^	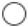	0.32 ± 0.01 ^c^	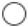	0.26 ± 0.02 ^c^	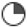	0.44 ± 0.03 ^d^	**<0.001**
***sirt2***	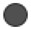	1.44 ± 0.12 ^a^	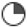	0.94 ± 0.06 ^b^	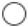	0.69 ± 0.03 ^c^	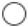	0.75 ± 0.03 ^c^	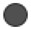	1.33 ± 0.08 ^a^	**<0.001**
***sirt3.1***	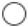	0.57 ± 0.03 ^a^	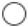	0.62 ± 0.06 ^b^	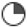	0.83 ± 0.03 ^ab^	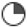	1.01 ± 0.07 ^b^	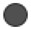	1.75 ± 0.09 ^c^	**<0.001**
***sirt3.2***	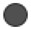	0.94 ± 0.05 ^a^	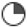	0.42 ± 0.05 ^b^	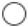	0.11 ± 0.01 ^c^	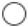	0.09 ± 0.01 ^c^	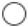	0.11 ± 0.01 ^c^	**<0.001**
***sirt3.1/3.2***	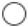	0.64 ± 0.06 ^a^	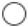	1.53 ± 0.11 ^a^	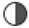	7.68 ± 0.43 ^b^	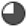	11.85 ± 0.36 ^c^	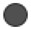	16.52 ± 0.89 ^d^	**<0.001**
***sirt5a***	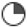	1.19 ± 0.14 ^a^	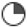	1.19 ± 0.11 ^a^	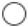	1.04 ± 0.09 ^a^	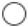	0.96 ± 0.09 ^a^	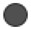	1.79 ± 0.21 ^b^	**0.002**
***sirt5b***	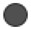	1.25 ± 0.15 ^a^	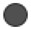	1.22 ± 0.23 ^a^	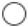	0.14 ± 0.01 ^b^	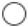	0.13 ± 0.02 ^b^	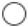	0.17 ± 0.01 ^b^	**<0.001**
***sirt5a/5b***	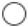	0.97 ± 0.11 ^a^	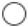	0.88 ± 0.11 ^a^	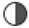	6.71 ± 0.57 ^b^	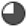	6.87 ± 0.43 ^b^	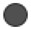	10.78 ± 1.20 ^c^	**<0.001**
***igfbp1a***	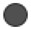	1.07 ± 0.09 ^a^	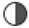	0.67 ± 0.06 ^b^	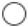	0.20 ± 0.02 ^c^	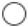	0.18 ± 0.02 ^c^	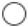	0.25 ± 0.02 ^c^	**<0.001**
***igfbp1b***	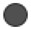	6.87 ± 2.66^a^	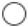	0.02 ± 0.00 ^b^	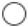	0.01 ± 0.01 ^b^	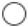	0.01 ± 0.01 ^b^	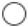	0.01 ± 0.00 ^b^	**<0.001**
***igfbp3a***	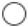	1.9 ± 0.15^a^	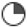	3.09 ± 0.22 ^ab^	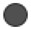	5.12 ± 0.56 ^b^	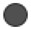	4.54 ± 0.61 ^b^	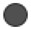	4.83 ± 0.55 ^b^	**0.013**
***igfbp3b***	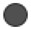	0.25 ± 0.04 ^a^	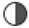	0.15 ± 0.02 ^b^	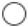	0.02 ± 0.01 ^c^	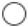	0.01 ± 0.00 ^c^	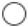	0.04 ± 0.03 ^c^	**<0.001**

**Table 2:** Relative gene expression of liver of gilthead sea bream across the development process. Data are the mean ± SEM of 6–10 fish. Expression values are referenced to the expression level of *sirt1* at 81 dph (days post-hatching), for which a value of 1 was assigned. *p*-values are the result of one-way analysis of variance and different superscript letters indicate significant differences (Holm–Sidak test, *p* < 0.05, bod). For each gene, gradation of black filling in circles indicates the expression levels along development.

		Liver							
		81 dph		127 dph		1 Year		3 Years	*p*-Value
***sirt1***	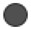	1.01 ± 0.07 ^a^	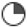	0.59 ± 0.05 ^b^	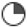	0.54 ± 0.04 ^b^	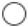	0.35 ± 0.02 ^c^	**<0.001**
***sirt2***	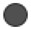	2.5 ± 0.18	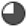	2.27 ± 0.19	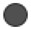	2.39 ± 0.17	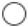	1.88 ± 0.10	**0.049**
***sirt3.1***		1.08 ± 0.08		0.99 ± 0.06		1.11 ± 0.09		0.94 ± 0.06	0.412
***sirt3.2***	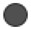	0.67 ± 0.06 ^a^	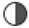	0.49 ± 0.06 ^b^	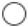	0.30 ± 0.03 ^c^	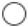	0.31 ± 0.02 ^c^	**<0.001**
***sirt3.1/3.2***	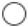	1.64 ± 0.11 ^a^	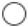	1.91 ± 0.15 ^a^	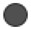	3.76 ± 0.29 ^b^	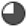	3.12 ± 0.24 ^b^	**<0.001**
***sirt5a***		2.61 ± 0.25		2.25 ± 0.1		2.36 ± 0.22		2.03 ± 0.11	0.214
***sirt5b***		1.28 ± 0.12		1.05 ± 0.07		1.07 ± 0.10		0.91 ± 0.10	0.142
***sirt5a/5b***		2.07 ± 0.15		2.16 ± 0.09		2.23 ± 0.23		2.35 ± 0.18	0.779
***igfbp1a***	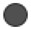	1.27 ± 0.35 ^a^	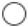	0.30 ± 0.04 ^b^	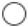	0.36 ± 0.04 ^b^	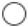	0.36 ± 0.02 ^b^	**<0.001**
***igfbp1b***	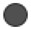	157.22 ± 20.61 ^a^	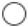	16.36 ± 4.80 ^b^	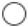	13.40 ± 1.37 ^b^	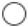	16.08 ± 4.80 ^b^	**<0.001**
***igfbp3a***		0.13 ± 0.05		0.08 ± 0.01		0.07 ± 0.01		0.08 ± 0.01	0.196
***igfbp3b***	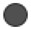	0.62 ± 0.34 ^a^	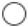	0.07 ± 0.03 ^b^	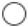	0.03 ± 0.00 ^b^	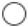	0.01 ± 0.00 ^b^	**0.014**

## Data Availability

Data of PhylomeDB evidencing up to three new gilthead sea bream *sirt* gene isotypes that were embraced by SIRT3 and SIRT5 branches (Figures S1 and S2) (http://phylomedb.org (accessed on 23 May 2022); PHY00CLOW5_SPAAU and PHY00CKZTK_SPAAU accession numbers). Novel *sirt* sequences were uploaded to GenBank with accession numbers OR394775 (*sirt3.1a*), OR394776 (*sirt3.1b*) and OR394777 (*sirt5b*), with their gilthead sea bream paralogs also renamed as *sirt3.2* (AHX56275, before *sirt3*) and *sirt5a* (AHX56277, before *sirt5*).

## Supplementary Materials

The following supporting information can be downloaded at: https://www.mdpi.com/article/10.3390/ijms25116273/s1.
