# Supplementary material for: The Expansion of Sirtuin Gene Family in Gilthead Sea Bream (Sparus aurata)—Phylogenetic, Syntenic, and Functional Insights across the Vertebrate/Fish Lineage
Source: Int J Mol Sci. 2024 Jun 6;25(11):6273. doi: 10.3390/ijms25116273 (PMC11172991; doi:10.3390/ijms25116273)
Supplement: Supplementary file 1 [file ijms-25-06273-s001.zip › Table S2. Primers gene expression.pdf]

Supplementary Table 2. Forward and reverse primers for the whole body, liver and white skeletal muscle pathway-focused qPCR array.

Gene name	Symbol		Primer sequence
Sirtuin1	*sirt1*	F	GGT TCC TAC AGT TTC ATC CAG CAG CAC ATC
		R	CCT CAG AAT GGT CCT CGG ATC GGT CTC
Sirtuin2	*sirt2*	F	GAA CAA TCC GAC GAC AGC AGT GAA G
		R	AGG TTA CGC AGG AAG TCC ATC TCT
Sirtuin3.1 (a+b)	*sirt3.1* (a+b)	F	CGG AGG AAC TAC AAG GGA GAG GAG
		R	GTC CCG CTC ATC ACA TCT GGT CG
Sirtuin3.2	*sirt3.2*	F	CTG CCA AGT CCT CAT CCC
		R	CTT CAC CAG ACG AGC CAC
Sirtuin4	*sirt4*	F	GGC TGG CGG AGT CGG ATG
		R	TCC TGA ATA CAC CTG TGA CGA AGA C
Sirtuin5a	*sirt5a*	F	CAG ACA TCC TAA CCC GAG CAG AG
		R	CCA CGA GGC AGA GGT CAC A
Sirtuin5b	*sirt5b*	F	CGG TCT ACG GAT GTG CTC CAC TAA TGC
		R	GTC TTG TTG TGT CCA CTA CAG GTC CTC TCA
Sirtuin6	*sirt6*	F	ACT CCA CCA CCA CCG ATG TCA A
		R	CTC CTC CTC CTT CAC CTT TCG CTT TG
Sirtuin7	*sirt7*	F	CTG GAG CAA CCT CTA AAC TGG AA
		R	CAC CTT CAG ACT GGA GCC TAA
Insulin-like growth factor binding protein 1a	*igfbp1a*	F	ACA AAC CAA AAC AGT GCG AGT CCT C
		R	CCG TTC CAA GAG TTC ACA CAC CAG
Insulin-like growth factor binding protein 1b	*igfbp1b*	F	GCC AAA CAG TGT GAG TCA TC
		R	ATC TTC TTC CCG TTC CAG G
Insulin-like growth factor binding protein 3a	*igfbp3a*	F	ACA GGC GTG TGG AGT GTA
		R	TGG TGC TGG CAG GTC AAG
Insulin-like growth factor binding protein 3b	*igfbp3b*	F	GCC AGA TTA TGG TCC CTG TCG GAG AGA G
		R	GTC TGT AAT CTT GAG GCT GCT GAG GAT GCT
rRNA 18S	*rRNA 18S*	F	GCA TTT ATC AGA CCC AAA ACC
		R	AGT TGA TAG GGC AGA CAT TCG
β-Actin	*actb*	F	TCC TGC GGA ATC CAT GAG A
		R	GAC GTC GCA CTT CAT GAT GCT
