# Supplementary material for: The Expansion of Sirtuin Gene Family in Gilthead Sea Bream (Sparus aurata)—Phylogenetic, Syntenic, and Functional Insights across the Vertebrate/Fish Lineage
Source: Int J Mol Sci. 2024 Jun 6;25(11):6273. doi: 10.3390/ijms25116273 (PMC11172991; doi:10.3390/ijms25116273)

Figure S2: Identification of SIRT5/*sirt5* genes in teleost and non-teleost species. The phylogenetic tree was constructed using phylomeDB v4 and the *sirt5* gene sequence from *Sparus aurata* as the seed. Red squares indicate branches in which duplication events are detected and speciation events are denoted with blue squares. PFAM motifs in the encoded proteins are presented at the right. Gap regions are illustrated as a flat line and standard amino acid colour codes are used for interdomain coding regions.

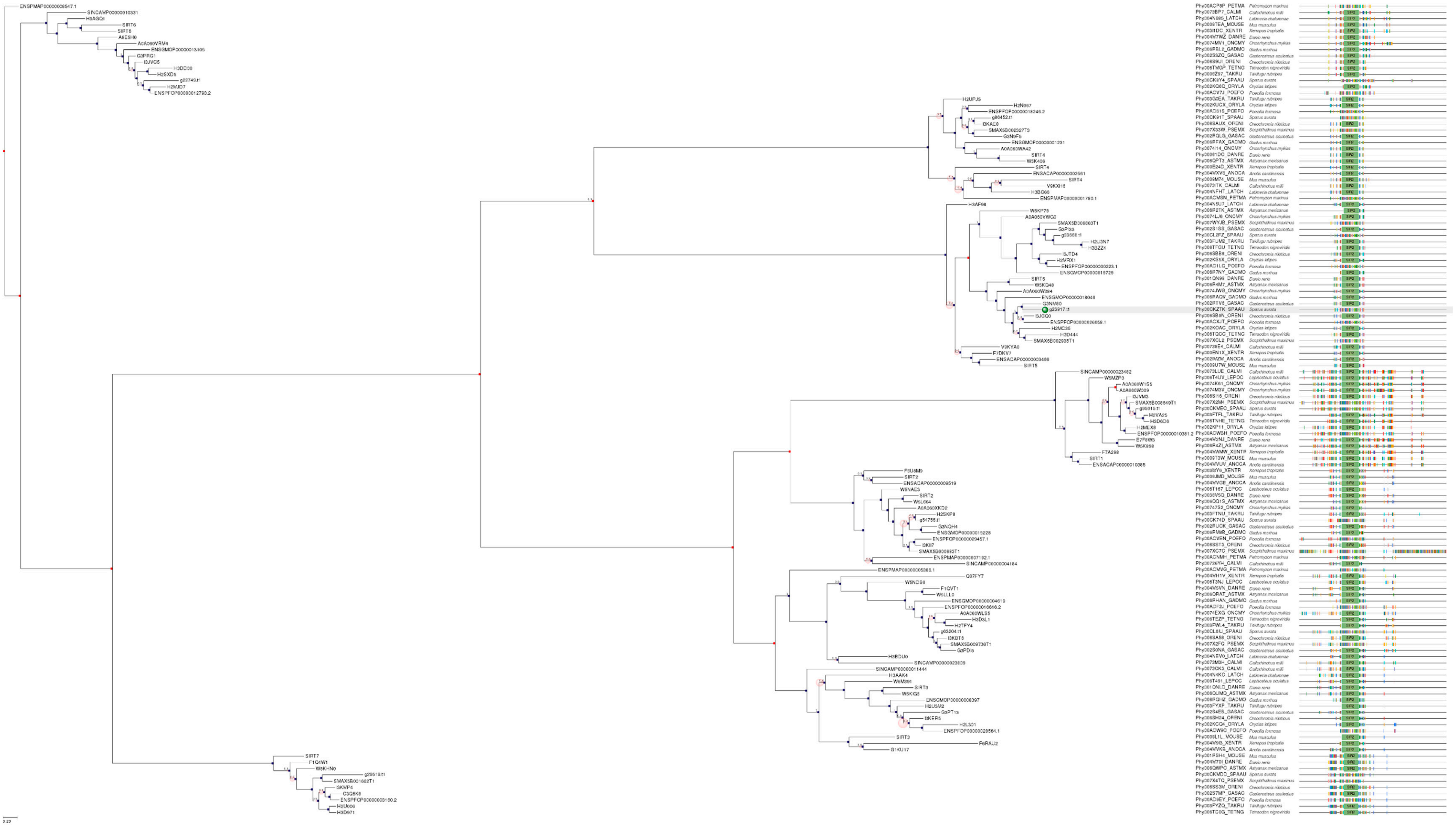

Supplement: Supplementary file 1 [file Figure S2.pdf › Figure S2.pdf]
